# Impact of Replacement Therapy on Pregnancy Outcomes in Hemophilia Carriers: A Historical Cohort Study in Saudi Arabia

**DOI:** 10.3390/life14050623

**Published:** 2024-05-11

**Authors:** Ebtisam Bakhsh

**Affiliations:** Internal Medicine Department, College of Medicine, Princess Nourah bint Abdulrahman University, Riyadh 11564, Saudi Arabia; ebtisam77@yahoo.com

**Keywords:** hemophilia carriers, replacement therapy, pregnancy outcomes, clotting factors, bleeding complications

## Abstract

This retrospective cohort study evaluates the safety and efficacy of replacement therapy with regard to pregnancy outcomes in hemophilia carriers. Hemophilia carriers face elevated bleeding risks during pregnancy, necessitating meticulous management, including replacement therapy with clotting factors. This research examines the records of 64 pregnant hemophilia carriers at King Fahad Medical City, Riyadh, from January 2010 to December 2023, analyzing their demographic details, hemophilia type and severity, replacement therapy specifics, and pregnancy outcomes. The study found that 62.5% of the participants had hemophilia A, with 43.8% categorized as severe. Most subjects (87.5%) received recombinant factor VIII at a median dosage of 30 IU/kg weekly. Adverse pregnancy outcomes included gestational hypertension (15.6%), preterm labor (18.8%), and postpartum hemorrhage (12.5%). The cesarean section rate was 28.1%. Neonatal outcomes were generally favorable, with median birth weights at 3100 g and mean Apgar scores of 8.2 and 9.1 at 1 and 5 min, respectively. Logistic regression analysis revealed no significant association between adverse events and therapy type or dosage, though a trend towards significance was noted with once-weekly administration (*p* = 0.082). The study concludes that replacement therapy is a viable method for managing hemophilia in pregnant carriers, leading to generally favorable maternal and neonatal outcomes. However, it underscores the importance of individualized treatment plans and close monitoring to effectively manage the risks associated with hemophilia during pregnancy.

## 1. Introduction

Hemophilia is a genetic bleeding disorder characterized by the inability of blood to clot properly, which leads to prolonged bleeding following injury or surgery [1,2]. The most common types of this disorder are hemophilia A and hemophilia B, distinguished primarily by the specific clotting factor deficient in the blood [3]. hemophilia A, the more prevalent of the two, involves a deficiency in factor VIII, while hemophilia B (also known as Christmas disease) is caused by a deficiency in factor IX [4,5,6]. Both types follow a similar pattern of symptoms, including spontaneous bleeding episodes, particularly into joints and muscles, but hemophilia A occurs approximately four times more frequently than hemophilia B [7].

The genetics of hemophilia are predominantly X-linked recessive, meaning the defective gene is located on the X chromosome [8]. This genetic pattern explains why males are more commonly affected when they inherit a defective gene [9]. Females, on the other hand, usually carry the defective gene without showing symptoms, making them carriers. A male’s X chromosome is transmitted to his daughters and the Y chromosome is transferred to his sons. If an affected male has a child with a healthy female, none of his male offspring will be affected, but all of his female offspring will be carriers, termed obligate carriers [10]. However, female carriers can experience symptoms if their other X chromosome fails to compensate—a condition that is not rare as once thought [11]. Recent studies suggest that up to 30% of carriers have some clotting challenges significant enough to be considered symptomatic. The prevalence of hemophilia varies globally, but approximately one in five to ten thousand males globally are born with hemophilia A, and around one in forty thousand with hemophilia B [12,13]. Estimates for female carriers are less precise but crucial for understanding the full impact of the disease, particularly during pregnancy, when both the carrier and the fetus may face increased risks [14,15,16].

Pregnancy in hemophilia carriers poses unique challenges due to the increased risk of bleeding, which can exacerbate the underlying condition [17]. While hemophilia primarily affects males, female carriers can experience varying degrees of bleeding symptoms, which may intensify during pregnancy due to hormonal changes and increased blood volume [18,19]. The most common complication is an enhanced risk of hemorrhage during delivery, which necessitates careful planning and monitoring. Additionally, carriers may encounter bleeding complications after invasive procedures such as amniocentesis, or even naturally occurring events such as miscarriage, which require specialized management to mitigate risks [20,21]. Beyond these acute events, carriers often face other pregnancy-related issues such as heavy menstrual bleeding and potential bleeding in the post-partum period, adding layers of complexity to both antenatal and postnatal care [22].

The impact of these bleeding complications extends beyond the maternal health and can significantly affect fetal outcomes [23]. Intrauterine growth restriction (IUGR) and preterm birth are potential risks, particularly if the carrier experiences significant bleeding or requires medical interventions during the pregnancy [24]. Moreover, the delivery method must be carefully chosen to minimize trauma and reduce the risk of hemorrhage for both mother and child. The possibility of transmitting the hemophilia gene to the offspring also adds an emotional and medical layer to the challenges, as there is a 50% chance that a male child will inherit the disorder if the mother is a carrier [19,25]. Thus, the management of hemophilia carriers during pregnancy is critical not only to safeguard the health of the mother but also to ensure the wellbeing of the newborn [26].

The management of hemophilia has significantly evolved over recent decades, largely due to advancements in prophylactic treatments and replacement therapies [27,28]. For non-pregnant patients, the cornerstone of hemophilia treatment involves regular administration of the deficient clotting factor, either as a preventive measure (prophylaxis) or to treat bleeding episodes when they occur (on-demand therapy) [29]. Prophylactic treatment has been shown to reduce the frequency of bleeding episodes and prevent joint damage, a common complication of recurrent bleedings [30,31]. Furthermore, recent developments in gene therapy hold promise for longer-term solutions by potentially correcting the underlying genetic defects that cause hemophilia. These approaches aim to maintain a steady level of clotting factor in the blood, thus mimicking the body’s natural way of preventing excessive bleeding [32,33,34].

During pregnancy, the management of hemophilia carriers or patients must be meticulously tailored to accommodate both the mother’s and the fetus’s needs [35]. The physiological changes that occur during pregnancy can alter clotting factor levels, sometimes increasing them, which may reduce bleeding symptoms but can also complicate the assessment and adjustment of prophylactic dosages [36,37]. Replacement therapy, involving the administration of clotting factors, remains a critical component during this time, especially during the third trimester, at delivery, and in the postpartum period to manage and prevent bleeding episodes [38,39]. The choice of factor concentrate is carefully considered to avoid complications such as the development of inhibitors (antibodies that can neutralize the effectiveness of replacement factors) and to ensure safety for both mother and child. Close monitoring by a multidisciplinary team of hematologists, obstetricians, and other specialists is essential to optimize outcomes and adapt treatment plans as pregnancy progresses [40].

Replacement therapy in the context of hemophilia involves the administration of clotting factors that are either deficient or defective in individuals with the disorder [41,42]. This treatment is pivotal in managing bleeding episodes and in prophylactic regimens to prevent bleeding. The two primary types of clotting factors used are factor VIII for hemophilia A and factor IX for hemophilia B, which are available in both plasma-derived and recombinant forms [43,44]. The introduction of recombinant clotting factors has significantly reduced the risk of transmitting blood-borne infections, a concern that existed with plasma-derived factors in the past. For pregnant hemophilia carriers, replacement therapy is critical during periods of increased bleeding risk, such as during labor and delivery and postpartum [45].

While replacement therapy is highly effective in managing hemophilia, it comes with its own set of challenges and risks, particularly during pregnancy. The dosages of clotting factors may need to be adjusted due to physiological changes in blood volume and clotting factor levels during pregnancy [46]. There is also a risk of developing inhibitors, antibodies that the body creates to work against the infused clotting factors, which can render the therapy ineffective. Additionally, the safety of continuous infusion or high doses during pregnancy needs careful evaluation to prevent complications such as thrombosis, ensuring that the benefits of preventing bleeding outweigh the potential risks [46,47,48].

The aim of this study was to systematically evaluate the effectiveness and safety of replacement therapy on pregnancy outcomes among hemophilia carriers. The goal was to improve evidence-based management strategies, enhancing care and health outcomes for both mothers and their children.

## 2. Materials and Methods

**Design:**

This retrospective study investigated the pregnancy outcomes in hemophilia carriers who underwent replacement therapy at King Fahad Medical City in Riyadh between January 2010 and December 2023. By examining the medical records of this single cohort, the study assessed the safety and effectiveness of replacement therapy, documenting specific outcomes such as the incidence of bleeding episodes, modes of delivery, and neonatal health status.

**Sample and Sampling:**

The study population consisted of female hemophilia carriers who were pregnant during the period from January 2010 to December 2023 and received medical care at King Fahad Medical City, Riyadh. From the hospital’s comprehensive database, a total of 1324 medical records of pregnant women were initially reviewed.

The selection criteria were specifically designed to include only those patients who had a confirmed diagnosis of being a carrier of hemophilia A or B and who underwent replacement therapy during their pregnancy. This included any form of clotting factor concentrate administered as part of their treatment regimen. Exclusion criteria were set to omit records with incomplete data regarding replacement therapy details or pregnancy outcomes, as well as those patients who received no replacement therapy during pregnancy, since the focus of this study was to observe outcomes linked directly to the intervention.

**Figure 1:**
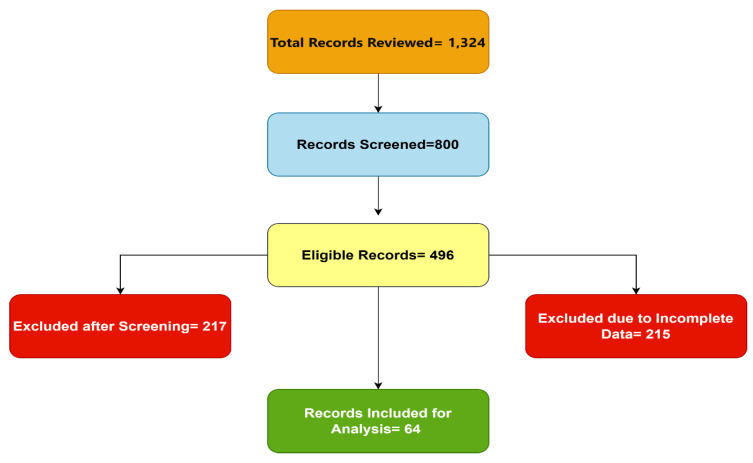
Participant selection flowchart for the retrospective study.

The flowchart (Figure 1) depicts the stages of participant selection for the study. Initially, a total of 1324 medical records were reviewed. Following this, 800 records were screened for potential eligibility. Out of these, 496 records were identified as eligible for inclusion in the study. At this juncture, the eligible records were further assessed, resulting in the exclusion of 217 records after screening, mainly due to them not meeting the specific study criteria. Additionally, 215 records were excluded due to incomplete data crucial for the study’s analysis. After this rigorous selection process, 64 records were deemed to have met all the inclusion criteria and contained complete data and were thus included for detailed analysis in the study.

### 2.1. Data Collection

#### 2.1.1. Data Collection Tool

The primary tool for data collection in this retrospective study was the hospital’s electronic health record (EHR) system. This comprehensive system houses detailed patient records, including demographics, medical history, diagnosis information, treatment details, and outcomes. For this study, a customized data extraction form was developed to systematically collect all relevant data points needed for the analysis. The extraction process involved the detailed and systematic recording of information. Each record was scrutinized for specific details relevant to the study’s objectives. The data extraction form served as the primary method for capturing data points directly from the EHR, ensuring that each element of patient history, treatment regimen, and outcomes was accurately logged. Research assistants filled out the form for each patient, ensuring that fields such as dates of treatment, types and amounts of administered clotting factors, and detailed pregnancy and delivery outcomes were comprehensively documented. The data collected included both quantitative measures, such as dosage levels, and qualitative descriptions, such as the nature of any complications.

#### 2.1.2. Data Collection Procedure

Data collection was conducted in several phases to ensure the accuracy and completeness of the extracted information. Initially, the researcher worked with hospital IT specialists to identify and retrieve all potential records from the EHR system based on the predefined inclusion and exclusion criteria. Once the records were identified, trained research assistants performed a manual review of each record using the data extraction form.

The researcher meticulously recorded each relevant data point into the form, ensuring consistency and precision in data capture. To maintain data integrity, a second member of the team reviewed a random sample of the forms to check for accuracy and completeness. Any discrepancies found during this verification process were resolved through discussion or re-review of the original medical records. This dual-check system minimized the risk of data entry errors and ensured a high level of data reliability for subsequent analysis.

### 2.2. Ethical Considerations

This retrospective study was conducted in strict accordance with ethical standards to ensure the protection of patient privacy and data integrity. Prior to data collection, approval was obtained from the Institutional Review Board (IRB) at King Fahad Medical City (H-01-R-059) in December 2023. All patient data accessed during the study were fully anonymized to safeguard patient identities, with personal identifiers removed before analysis. The study adhered to the principles of the Declaration of Helsinki, ensuring that all research practices were conducted ethically and responsibly.

### 2.3. Data Analysis

The data analysis for this retrospective cohort study was conducted using statistical software (SPSS) (version 26) to assess the effectiveness and safety of replacement therapy in pregnant hemophilia carriers. Descriptive statistics were first applied to summarize the demographics and clinical characteristics of the study participants. Comparative analyses were then performed using chi-square tests for categorical variables and *t*-tests for continuous variables to compare outcomes between those who received replacement therapy and those who did not. Logistic regression models were employed to control for potential confounders and assess the impact of replacement therapy on pregnancy outcomes. A *p*-value of less than 0.05 was considered statistically significant, indicating a meaningful difference in the outcomes attributed to the therapy. This comprehensive statistical approach allowed for a robust evaluation of the data, providing clear insights into the correlations between replacement therapy and pregnancy outcomes in hemophilia carriers.

## 3. Results

Table 1 provides overview of the demographic and baseline characteristics of the study participants, consisting of 64 individuals. It includes information on age, body mass index (BMI), type of hemophilia carrier, severity of hemophilia, history of bleeding episodes, number of previous pregnancies, and number of previous live births. The mean age of participants was 28.5 years, with a range of 18 to 40 years, and the mean BMI was 26.3 kg/m^2^, ranging from 18.5 to 34.6 kg/m^2^. The majority of participants were type A hemophilia carriers (62.5%), with the remaining 37.5% being type B carriers. Regarding hemophilia severity, 23.4% had mild, 32.8% had moderate, and 43.8% had severe hemophilia. Most participants (75%) had a history of bleeding episodes. On average, participants had had 1.2 previous pregnancies and 0.8 previous live births, with ranges of 0–4 and 0–3, respectively.

Table 2 offers a concise summary of the hemophilia diagnosis and carrier status among participants, showcasing the distribution of confirmed and possible carriers across hemophilia types and severities. For instance, it reveals that among the confirmed carriers, the majority exhibited moderate hemophilia A (18 participants), followed closely by mild hemophilia A (12 participants). Severe cases were relatively less common, with nine participants diagnosed with severe hemophilia A and three with severe hemophilia B. Conversely, among the possible carriers, mild hemophilia A was the most prevalent, accounting for four participants, while other severities and hemophilia types showed lower or no representation.

Table 3 provides an overview of replacement therapy details regarding the pregnant hemophilia carriers. It includes information on the type of replacement therapy used, with 87.5% receiving recombinant factor VIII and 12.5% receiving plasma-derived factor VIII. Dosage and frequency of administration are detailed, with a median dosage of 30 IU/kg per week and various frequencies reported, such as once weekly (18.8%), twice weekly (28.1%), and three times weekly (15.6%). The median duration of therapy was 28 weeks. Reasons for replacement therapy use are highlighted, with 65.6% for bleeding episodes management, 12.5% for surgery preparation, and 21.9% for prophylactic use during pregnancy. Additionally, the table indicates instances of therapy switches, with 3.1% transitioning from recombinant to plasma-derived therapy and 9.4% vice versa.

Table 4 provides an overview of pregnancy, delivery, and neonatal outcomes among the pregnant hemophilia carriers. In terms of pregnancy outcomes, gestational hypertension and preterm labor were observed in 15.6% and 18.8% of cases, respectively, while gestational diabetes affected 9.4% of pregnancies. Postpartum hemorrhage occurred in 12.5% of deliveries. Cesarean section was the mode of delivery in 28.1% of cases, with vaginal delivery accounting for the majority at 71.9%. The median length of hospital stay was 4 days (IQR: 3–6). Neonatal outcomes revealed a median birth weight of 3100 g (IQR: 2800–3400) and mean Apgar scores of 8.2 at 1 min and 9.1 at 5 min. Neonatal intensive care admission was required for 7.8% of newborns.

Table 5 provides a detailed analysis of adverse events associated with replacement therapy in pregnant hemophilia carriers, coupled with logistic regression results. While the type and dosage of therapy show no significant association with adverse events, the frequency of administration, particularly once-weekly use, displays a trend towards significance (*p* = 0.082). Reasons for therapy use, such as surgery preparation, exhibit elevated odds ratios (*p* = 0.158), suggesting a potential association with adverse events. Conversely, prophylactic therapy during pregnancy shows no significant relationship with adverse events. Transitioning between therapy types also does not significantly impact adverse event occurrence.

Table 6 presents a comparison of pregnancy and neonatal outcomes between first-time and multigravida hemophilia carriers. Notably, the incidences of gestational hypertension, preterm labor, postpartum hemorrhage, and neonatal intensive care admission show minimal variation between first-time pregnancies and those who have had multiple pregnancies. The *p*-values indicate no significant differences in these outcomes, suggesting that the number of prior pregnancies does not dramatically affect these specific complications in hemophilia carriers. Similarly, cesarean section rates were slightly higher in multigravida carriers (36.0%) compared to first-time carriers (30.8%), yet this difference was not statistically significant (*p* = 0.60).

The birth weights and Apgar scores, which serve as indicators of neonatal health, also did not differ significantly between the two groups, supporting the notion that the level of neonatal care required at birth is comparable regardless of whether the pregnancy was a woman’s first or subsequent.

## 4. Discussion

The findings of this retrospective cohort study provide valuable insights into the utilization and outcomes of replacement therapy among pregnant hemophilia carriers. The study highlights the diverse treatment approaches employed, including the types of clotting factor concentrates used, dosing regimens, and indications for therapy. Furthermore, it sheds light on the intricate interplay between replacement therapy and pregnancy outcomes, adverse events, and neonatal wellbeing.

One of the key observations from the study was the predominance of recombinant factor VIII (87.5%) as the preferred choice for replacement therapy, aligning with contemporary treatment guidelines and practices [49,50]. The transition from plasma-derived to recombinant products has been a significant advancement in hemophilia management, reducing the risk of transmitting blood-borne pathogens and offering improved safety profiles [51]. This trend is reflected in the study population, as only a small proportion (12.5%) received plasma-derived factor VIII. However, it is noteworthy that in some cases, switching between recombinant and plasma-derived products occurred, potentially due to clinical considerations, availability, or patient preferences [52,53].

The dosing and frequency of administration varied considerably among the participants, ranging from once-weekly to thrice-weekly infusions, with a median dosage of 30 IU/kg per week. This variability highlights the individualized nature of treatment regimens, which are tailored to each patient’s unique clinical profile, bleeding risk, and response to therapy [54]. While the study did not find a significant association between dosage and adverse events, the frequency of administration, particularly once-weekly infusions, showed a trend towards significance (*p* = 0.082). This finding aligns with previous research suggesting that more frequent dosing may be associated with better prevention of bleeding episodes and improved overall outcomes [55,56].

The reasons for initiating replacement therapy also varied, with the majority (65.6%) receiving treatment for the management of bleeding episodes, followed by prophylactic use during pregnancy (21.9%) and preparation for surgery (12.5%). Interestingly, the logistic regression analysis revealed a trend towards increased odds of adverse events when replacement therapy was initiated for surgery preparation (OR = 1.57, *p* = 0.158). This observation underscores the potential challenges and risks associated with invasive procedures in hemophilia carriers, necessitating careful consideration and planning during replacement therapy [57,58].

Regarding pregnancy outcomes, the study documented a range of complications, including gestational hypertension (15.6%), preterm labor (18.8%), gestational diabetes (9.4%), and postpartum hemorrhage (12.5%). While these rates are consistent with those reported in the general population [59], the potential impact of hemophilia carrier status and replacement therapy on these outcomes warrants further investigation. Notably, the cesarean section rate (28.1%) was higher than the global average, which may reflect the complex management and potential bleeding risks associated with vaginal deliveries in hemophilia carriers [60,61,62]. It should be noted that the early diagnosis of pregnancy allows for timely initiation of prophylactic therapy, which is crucial for managing bleeding risks. Furthermore, among our cohort, a small subset of patients underwent IVF, reflecting the broader trend of assisted reproductive technologies in hemophilia carriers [63]

Neonatal outcomes, as assessed by birth weights, Apgar scores, and neonatal intensive care unit (NICU) admission rates, were generally favorable in this cohort. The median birth weight of 3100 g and mean Apgar scores of 8.2 (at 1 min) and 9.1 (at 5 min) suggest that replacement therapy played a role in maintaining favorable intrauterine conditions and minimizing the impact of potential bleeding complications on fetal development. However, it is important to note that 7.8% of newborns required NICU admission, highlighting the need for close monitoring and specialized care for these high-risk pregnancies [64,65].

While the study did not find a significant association between the type of replacement therapy (recombinant or plasma-derived) and adverse events, ongoing research is needed to evaluate the long-term safety and efficacy of different clotting factor concentrates in the context of pregnancy. The development of inhibitors, antibodies that neutralize the activity of replacement factors, remains a concern in hemophilia management, particularly during pregnancy when immune system changes may increase the risk [66]. Additionally, the potential for thrombotic events associated with high-dose or continuous infusion regimens requires careful consideration and monitoring [67].

### 4.1. Implications of the Study

The findings of this study have several important implications for clinical practice and future research. Firstly, it emphasizes the need for individualized treatment regimens for pregnant hemophilia carriers, considering factors such as the severity of the condition, bleeding risk, and response to therapy. The variability in dosing and frequency of administration observed in the study underscores the importance of tailoring replacement therapy to each patient’s unique clinical profile. Secondly, the study highlights the potential risks associated with invasive procedures, such as surgery, in hemophilia carriers, suggesting the need for careful planning and consideration during replacement therapy in these situations. Furthermore, the trend towards increased adverse events with once-weekly dosing frequencies raises questions about the optimal dosing interval for prophylactic treatment during pregnancy, an area that warrants further investigation.

### 4.2. Limitations of the Study

While this study provides valuable insights, it is essential to acknowledge its limitations. As a retrospective cohort study, it relies solely on the accuracy and completeness of medical record data, which may be subject to documentation biases or missing information. Additionally, the single-center design and relatively small sample size limit the generalizability of the findings to other populations or healthcare settings. Prospective, multicenter studies with larger cohorts would be necessary to confirm and expand upon these results. Furthermore, the study did not extensively explore long-term maternal and neonatal outcomes, which are crucial for a comprehensive understanding of the impact of replacement therapy during pregnancy.

## 5. Conclusions

This retrospective cohort study offers essential insights into the management of pregnant hemophilia carriers treated with replacement therapy at King Fahad Medical City. Analyzing 64 cases, we found that the predominant use of recombinant factor VIII leads to generally favorable outcomes for both mothers and newborns. Despite the inherent challenges of managing hemophilia during pregnancy, our findings support the efficacy and safety of this therapeutic approach. Notably, the study did not identify significant correlations between adverse events and the type or dosage of therapy used.

However, our analysis suggests the necessity of vigilant, continuous monitoring and highly individualized treatment plans, particularly as there was a noticeable trend toward significance in adverse events associated with less frequent dosing during therapy. These findings emphasize the critical need for tailored healthcare strategies that cater specifically to the unique requirements of hemophilia carriers during pregnancy to ensure the safety of both mother and child.

Moreover, the study underlines the importance of further research to refine treatment protocols and investigate the long-term impacts of replacement therapy on this patient population. By enhancing our understanding and management strategies, we aim to improve overall outcomes for hemophilia carriers and their offspring during the demanding circumstances of pregnancy, highlighting the indispensable role of specialized, attentive care throughout this critical period.

## Figures and Tables

**Table 1:** Demographic and baseline characteristics of study participants.

Variable		Total Cohort (n = 64)
Age (years)	- Mean (SD)	28.5 (±5.7)
	- Range	18–40
Body Mass Index (BMI) (kg/m^2^)	- Mean (SD)	26.3 (±4.2)
	- Range	18.5–34.6
Type of Hemophilia Carrier	- Type A (n, %)	40 (62.5%)
	- Type B (n, %)	24 (37.5%)
Severity of Hemophilia	- Mild (n, %)	15 (23.4%)
	- Moderate (n, %)	21 (32.8%)
	- Severe (n, %)	28 (43.8%)
History of Bleeding Episodes	- Yes (n, %)	48 (75%)
	- No (n, %)	16 (25%)
Number of Previous Pregnancies	- Mean (SD)	1.2 (±1.3)
	- Range	0–4
Number of Previous Live Births	- Mean (SD)	0.8 (±1.1)
	- Range	0–3

**Table 2:** Details of hemophilia diagnosis and carrier status of participants.

Carrier Status	Hemophilia Type	Severity	Number of Participants
Confirmed Carrier	Type A	Mild	12
Confirmed Carrier	Type A	Moderate	18
Confirmed Carrier	Type A	Severe	9
Confirmed Carrier	Type B	Mild	5
Confirmed Carrier	Type B	Moderate	10
Confirmed Carrier	Type B	Severe	3
Possible Carrier	Type A	Mild	4
Possible Carrier	Type A	Moderate	2
Possible Carrier	Type A	Severe	1
Possible Carrier	Type B	Mild	0
Possible Carrier	Type B	Moderate	0
Possible Carrier	Type B	Severe	0

**Table 3:** Replacement therapy details in pregnant hemophilia carriers.

Treatment Characteristics	Frequency n = 64	%
Type of Replacement Therapy		
Plasma derived factor VIII	8	12.5
Recombinant factor VIII	56	87.5
Dosage (IU/kg per week)		
Median (IQR)	30 (25–40)	
Frequency of Administration		
Once weekly	12	18.8
Twice weekly	18	28.1
Three times weekly	10	15.6
Other	24	37.5
Duration of Therapy (weeks)		
Median (IQR)	28 (24–32)	
Reason for Replacement Therapy Use		
Bleeding episodes management	42	65.6
Surgery preparation	8	12.5
Prophylactic during pregnancy	14	21.9
Switch of Therapy		
From recombinant to plasma derived	2	3.1
From plasma derived to recombinant	6	9.4

**Table 4:** Pregnancy, delivery, and neonatal outcomes.

Outcome		Frequency (n = 64)	Percentage (%)
Pregnancy Outcomes	Gestational hypertension	10	15.6
	Gestational diabetes	6	9.4
	Preterm labor	12	18.8
	Postpartum hemorrhage	8	12.5
	Cesarean section	18	28.1
	Vaginal delivery	46	71.9
Delivery Outcomes	Mode of delivery		
	- Vaginal	46	71.9
	- Cesarean section	18	28.1
	Length of hospital stay (days)		
	- Median (IQR)	4 (3–6)	
Neonatal Outcomes	Birth weight (g)		
	- Median (IQR)	3100 (2800–3400)	
	Apgar score at 1 min (mean ± SD)		
	- Mean ± SD	8.2 ± 1.0	
	Apgar score at 5 min (mean ± SD)		
	- Mean ± SD	9.1 ± 0.6	
	Neonatal intensive care admission	5	7.8

**Table 5:** Adverse events associated with replacement therapy and logistic regression analysis.

Treatment Characteristic	Adverse Events (n = 64)	Odds Ratio (95% CI)	*p*-Value
Type of Replacement Therapy			
- Plasma-derived Factor VIII	6 (9.4%)	1.35 (0.76–2.41)	0.288
- Recombinant Factor VIII	42 (65.6%)	0.87 (0.54–1.41)	0.562
Dosage (IU/kg per week)			
- Median (IQR)	30 (25–40) IU/kg/week	1.02 (0.98–1.05)	0.364
Frequency of Administration			
- Once weekly	12 (18.8%)	1.14 (0.98–1.33)	0.082
- Twice weekly	18 (28.1%)		
- Three times weekly	10 (15.6%)		
- Other	24 (37.5%)		
Duration of Therapy (weeks)			
- Median (IQR)	28 (24–32) weeks	0.98 (0.93–1.03)	0.512
Reason for Therapy Use			
- Bleeding episodes management	42 (65.6%)	Reference	-
- Surgery preparation	8 (12.5%)	1.57 (0.84–2.95)	0.158
- Prophylactic during pregnancy	14 (21.9%)	0.92 (0.55–1.55)	0.762
Switch of Therapy			
- From recombinant to plasma-derived	1 (1.6%)	1.23 (0.45–3.38)	0.678
- From plasma-derived to recombinant	3 (4.7%)	0.98 (0.58–1.65)	0.928

**Table 6:** Comparison of outcomes between first-time and multigravida hemophilia carriers.

Outcome	First-Time Pregnancy (n = 39)	Multiple Pregnancies (n = 25)	*p*-Value
Gestational Hypertension	5 (12.8%)	3 (12.0%)	0.95
Preterm Labor	6 (15.4%)	4 (16.0%)	0.92
Postpartum Hemorrhage	4 (10.3%)	3 (12.0%)	0.83
Cesarean Section	12 (30.8%)	9 (36.0%)	0.60
Neonatal Intensive Care Admission	3 (7.7%)	2 (8.0%)	0.96
Median Birth Weight (grams)	3100 (IQR: 2850–3350)	3000 (IQR: 2800–3200)	0.35
Apgar Score at 1 Min (mean ± SD)	8.2 ± 0.9	8.3 ± 0.8	0.75
Apgar Score at 5 Min (mean ± SD)	9.1 ± 0.6	9.2 ± 0.5	0.67

## Data Availability

Data available upon request.
